# Erythrodermic Psoriasis in a Man with Monoclonal B-cell Lymphocytosis

**DOI:** 10.7759/cureus.1936

**Published:** 2017-12-11

**Authors:** Stella X Chen, Brian R Hinds, Aaron M Goodman, Philip R Cohen

**Affiliations:** 1 School of Medicine, University of California, San Diego; 2 Department of Dermatology, University of California, San Diego; 3 Division of Hematology and Oncology, Division of Blood and Marrow Transplantation, University of California, San Diego

**Keywords:** b-cell, chronic, leukemia, lymphocytic, monoclonal, pilaris, pityriasis, psoriasis, rubra, erythroderma

## Abstract

Erythroderma is characterized by erythema involving greater than 90% of the body surface area and may be caused by several etiologies, including erythrodermic psoriasis. Psoriasis is an autoimmune skin and systemic condition characterized by erythematous and scaly plaques. Monoclonal B-cell lymphocytosis is an asymptomatic hematological disorder diagnosed by elevated, small, clonal B-cell counts in the peripheral blood. The characteristics of a 71-year-old man with new onset of erythrodermic psoriasis and concurrent monoclonal B-cell lymphocytosis are presented. The simultaneous development of these two conditions raises the possibility that they may share a related pathogenesis.

## Introduction

Erythroderma is a cutaneous disorder that manifests with partial to near confluent erythema involving greater than 90% of the body surface area [1]. It is associated with many underlying etiologies, including psoriasis. Psoriasis is an immune-mediated skin disorder that is most commonly characterized by well-marginated erythematous, scaly plaques [2]. Monoclonal B-cell lymphocytosis is defined as the presence of a clonal B-cell population in the peripheral blood with an absolute B-lymphocyte count of less than 5 x 10^9^/liter and no evidence of an associated lymphoproliferative disorder; however, it may be a precursor to chronic lymphocytic leukemia [3]. The case of a 71-year-old man presenting with erythrodermic psoriasis and concurrent monoclonal B-cell lymphocytosis is described herein.

## Case presentation

A 71-year-old man initially presented with a diffuse erythematous, exfoliative, and itchy rash on the scalp and ears concerning for seborrheic dermatitis. The desquamating rash progressed in a cephalocaudal manner with subsequent involvement of the palms, soles, and genitalia. Initial skin biopsy showed spongiotic dermatitis, and the patient was started on sequential treatments of systemic prednisone, cyclosporine, and narrow-band ultraviolet B therapy. Azathioprine was subsequently added due to lack of improvement.

Four months later, the patient underwent coronary artery stenting during which the azathioprine was discontinued. Upon re-initiation of azathioprine, he developed shortness of breath, fever, and worsening of his rash. He was diagnosed with a severe allergic reaction to azathioprine; the respiratory symptoms and fever promptly resolved with systemic corticosteroids.

**Figure 1:**
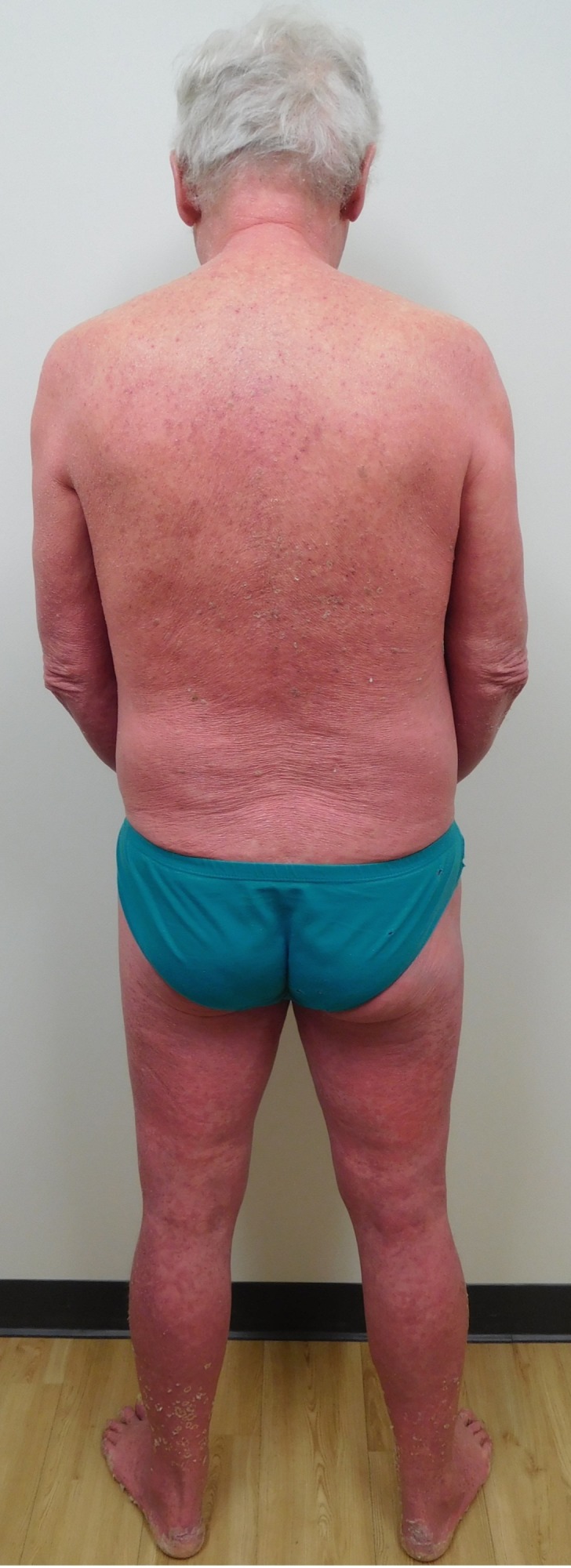
Psoriasis presenting as erythroderma The back and posterior legs of a 71-year-old Caucasian man with erythroderma presenting as red skin involving 92% of his body surface area.

**Figure 2:**
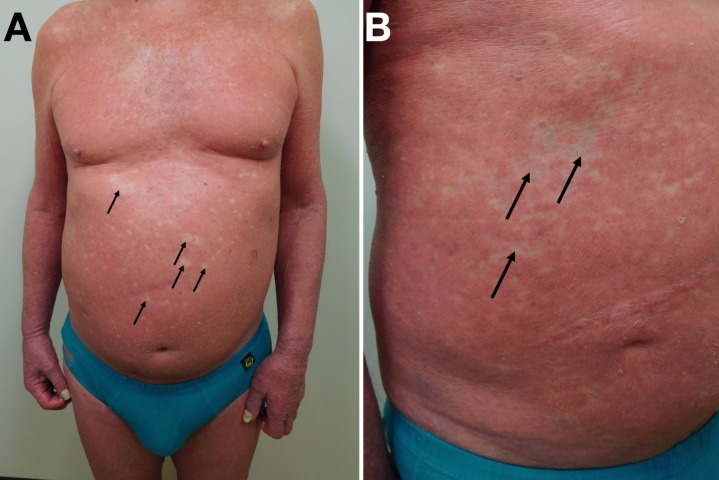
Erythrodermic psoriasis Distant (A) and closer (B) views of the chest and abdomen show confluent erythema with focal areas of uninvolved, normal-appearing skin (arrows), also known as “islands of sparing,” in a 71-year-old Caucasian man.

**Figure 3:**
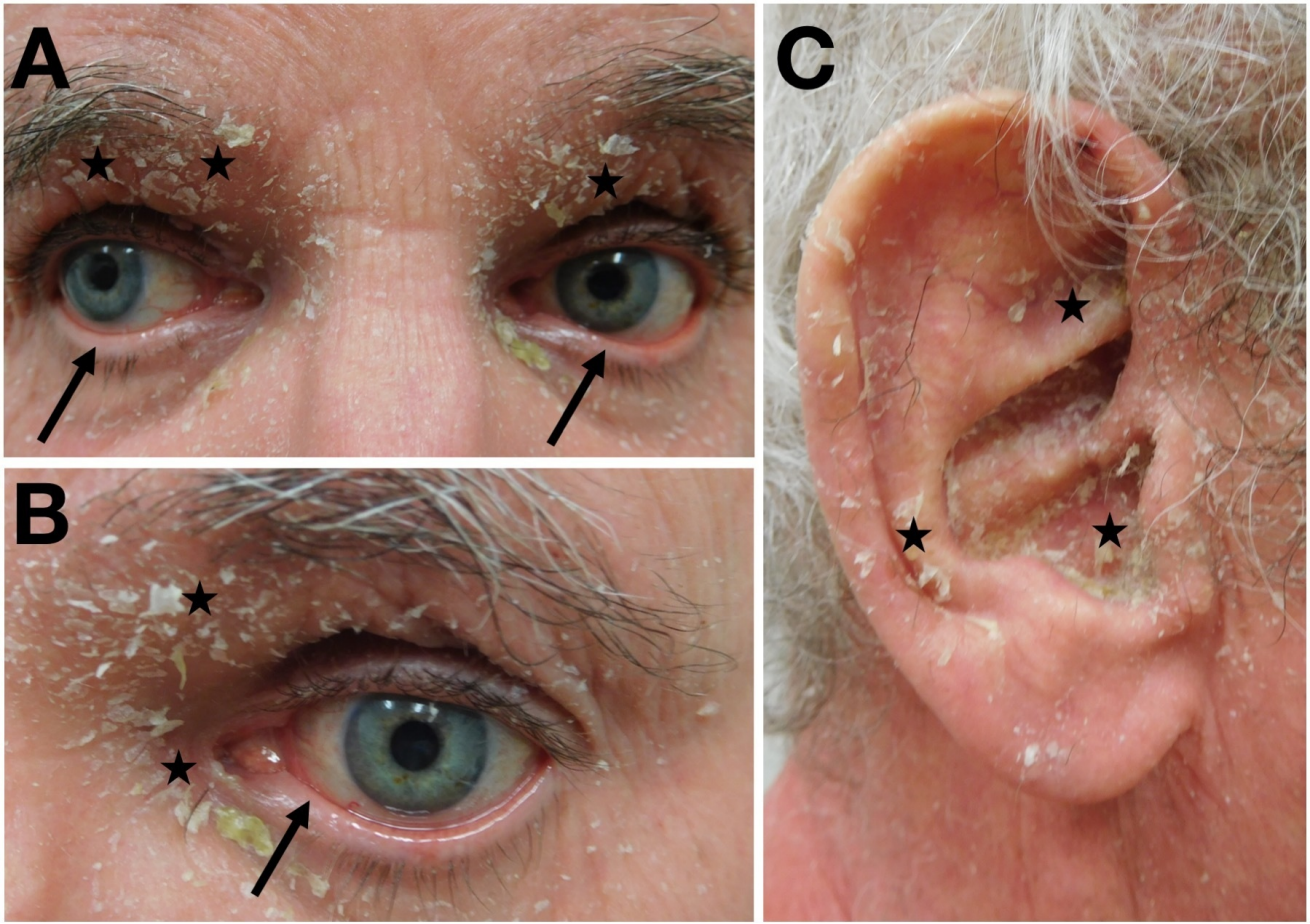
Erythrodermic psoriasis affecting the eyes and ears Distant (A) and closer (B and C) views of scaling and desquamation (stars) of the eyes (A and B) and the right ear (C); there are bilateral ectropions (arrows) of the lower eyelids.

**Figure 4:**
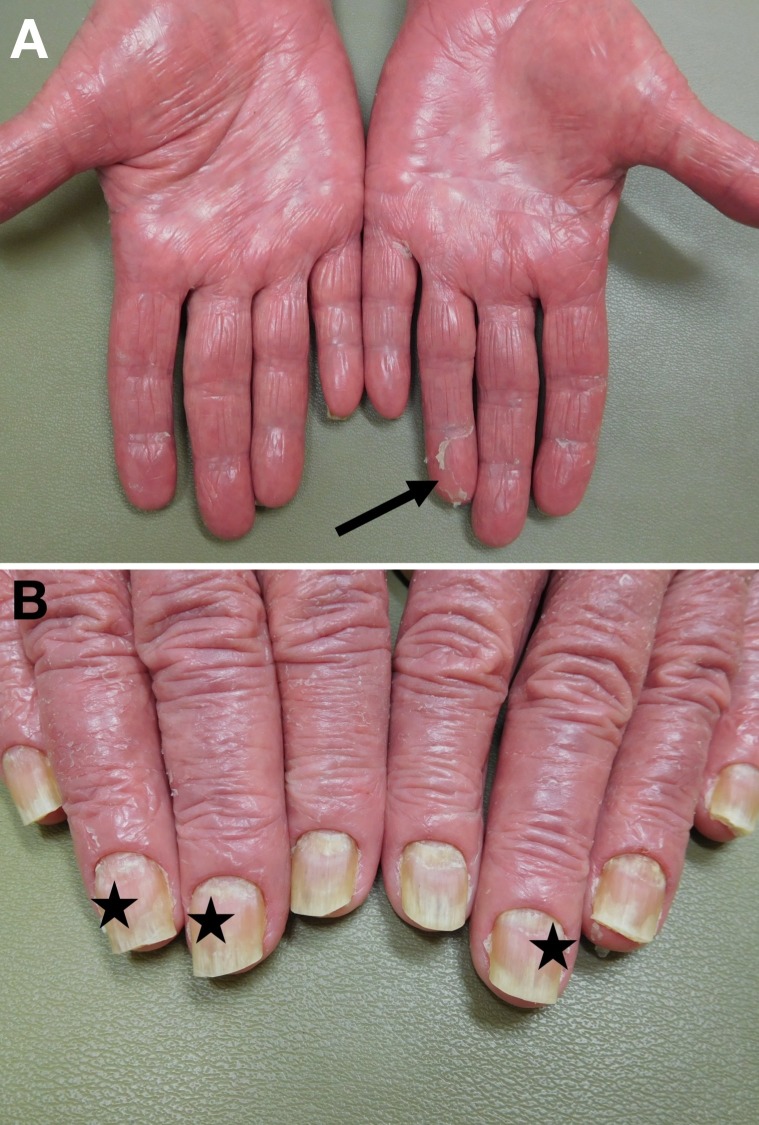
Erythrodermic psoriasis of the palms and fingernails Thickened, shiny, erythematous to orange palms (A) with desquamation around the digits (arrows) and proximal nail plate shedding with distal onycholysis (stars) (B) of both hands.

**Figure 5:**
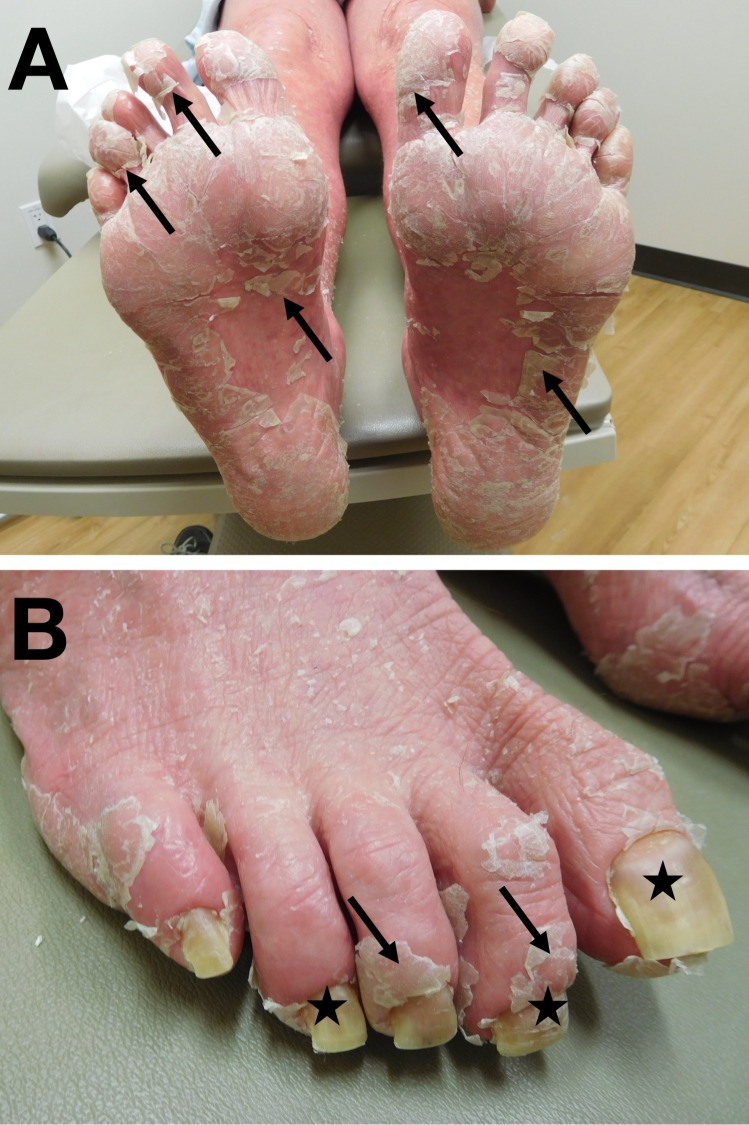
Erythrodermic psoriasis affecting the feet and toenails Desquamation of large flakes of skin (arrows) of the plantar feet (A) and dorsal toes (B) with proximal nail plate shedding and distal onycholysis (stars) (B).

However, five months after his initial presentation, the patient’s pruritic, desquamating, erythematous eruption continued to worsen and soon involved 92% of his body surface area with focal areas of normal, uninvolved skin with surrounding erythema most prominent on the abdomen (Figures 1-2). There were fine scaly plaques on the ears and eyes, and bilateral conjunctival injection and ectropion were seen (Figure 3). There were no oral findings. The palms were thickened, shiny, and confluently erythematous with desquamation and proximal shedding of the nails (Figure 4). Lateral and plantar feet demonstrated shedding of large pieces of skin with proximal shedding of the nails (Figure 5).

**Figure 6:**
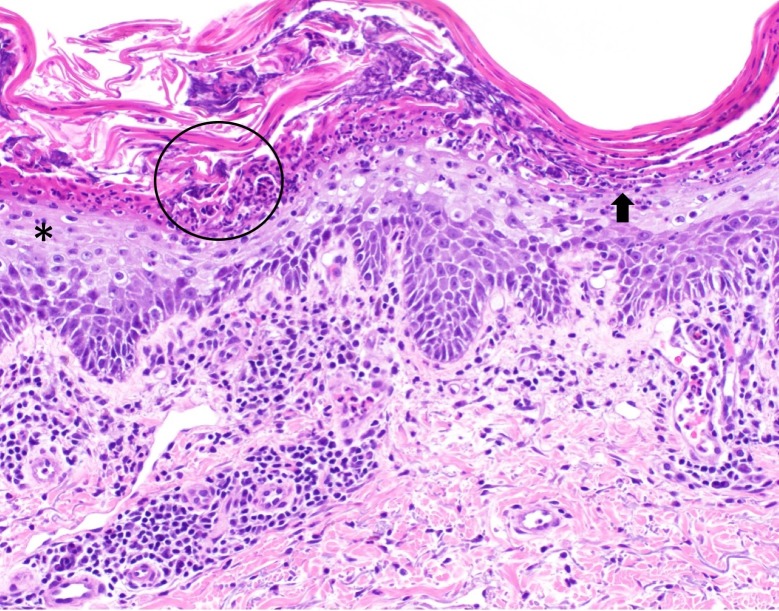
Pathologic features of erythrodermic psoriasis from a right upper chest skin biopsy Pronounced mounds of neutrophils cap an abnormal epithelial zone (circle) that demonstrates pallor of the spinous layer (asterisk), a psoriasiform contour, and reduction in the granular zone (arrow). A patchy lymphocytic infiltrate falls below this.Hematoxylin and eosin, 20X.

At this time, three punch biopsies were performed, two of which revealed parakeratosis with underlying pallid keratinocytes, subjacent neutrophils forming conical pustules, and a perivascular lymphocytic infiltrate (Figure 6). These findings were most suggestive of psoriasis. The patient’s clinical features and skin pathology were compatible with erythrodermic psoriasis. The third biopsy only showed a sparse superficial perivascular lymphocytic infiltrate.

A workup to exclude cutaneous T-cell lymphoma and other paraneoplastic syndromes was also initiated after the condition had been present for six months. T-cell receptor gene rearrangement studies showed no evidence of T-cell clonality; however, peripheral blood flow cytometry revealed a monotypic CD5+, CD19+, CD20+, and CD23+ lambda-restricted B-cell population. Subsequent bone marrow biopsy revealed no morphologic evidence of an underlying lymphoproliferative disorder. Flow cytometry on the bone marrow aspirate identified the same monotypic B-cell population. Computed tomography scan of the patient’s neck did not identify any lymphadenopathy. The patient was evaluated by hematology and diagnosed with low count monoclonal B-cell lymphocytosis.

Correlation of the patient’s clinical presentation and pathological findings established the diagnosis of erythrodermic psoriasis in a man with monoclonal B-cell lymphocytosis. After discussing the risks and benefits of systemic agents to treat his psoriasis (including methotrexate, retinoids, and biological agents), adalimumab (40 milligrams subcutaneous injection every two weeks) was selected for therapeutic intervention. At the six-week follow-up, the patient demonstrated mild improvement in his erythroderma, pruritus, and associated fatigue. However, these results were not satisfactory and the decision to switch to ustekinumab was made.

## Discussion

Erythroderma is a generalized erythema involving more than 90% of the body surface and may be exfoliative, exudative, and involve hair and nail changes [1]. Intense erythema always precedes desquamation, the latter of which reflects rapid epidermopoiesis. It has several etiologies, including infection, inflammatory skin conditions, malignancy, and systemic drug reactions (Table 1) [1, 4]. Our patient presented with several of the morphologic features classically seen in pityriasis rubra pilaris, an inflammatory skin disorder that can be causative of erythroderma. Interestingly, the IL12/23 pathway is a potential source for mechanistic overlap between the two seemingly disparate conditions, as IL12/23 blockade can curiously lead to a complete response for pityriasis rubra pilaris and psoriatic patients [5].

**Table 1:** Common Etiologies of Erythroderma ^1^This list includes many drugs commonly associated with erythroderma but is not complete.

Etiology and causes
Drug reactions^1^
Allopurinol
Amiodarone
Calcium-channel blockers
Cimetidine
Isosorbide dinitrate
Lithium
Omeprazole
Quinidine
St. John's Wort
Thiazides
Hematologic dyscrasias and malignancies
Langerhans cell histiocytosis
Lung carcinoma
Lymphoma (Anaplastic large cell lymphoma, diffuse large B-cell lymphoma)
Monoclonal B-cell lymphocytosis
Mycosis fungoides
Sezary syndrome
Tongue carcinoma
Infections
Dermatophytosis
Human immunodeficiency virus
Scabies
Inflammatory skin and autoimmune disorders
Actinic reticuloid
Atopic dermatitis
Bullous pemphigoid
Contact dermatitis
Dermatomyositis
Erythema toxicum neonatorum
Graft-versus-host disease
Idiopathic hypereosinophilic syndrome
Lichen planus
Pemphigus foliaceus
Psoriasis
Pityriasis rubra pilaris
Sarcoidosis
Seborrheic dermatitis
Systemic lupus erythematous

Pityriasis rubra pilaris is characterized by scaling and thickened patches of red-orange skin that may generalize to the entire body [6]. As in our patient, individuals with pityriasis rubra pilaris may develop a seborrheic dermatitis-like rash on the scalp that then progresses caudally. The scale on the face and scalp is often fine, while desquamation of the lower extremities is thicker. The erythroderma is often exfoliative and may be pruritic, containing focal areas of uninvolved skin known as “islands of sparing” [7]. The palms and soles are frequently involved and become orange and hyperkeratotic with thickened nail plates and proximal nail shedding. Ocular changes include ectropion, ulcerative keratitis, and corneal perforation, as well as dry eyes [6-7]. Many of these morphological features were also demonstrated in our patient.

Despite clinical similarities to pityriasis rubra pilaris, our patient’s erythroderma was further qualified with microscopic features indicative of erythrodermic psoriasis in a total of two of three skin biopsies. This highlights the necessary role of skin biopsies in an erythrodermic patient to establish the diagnosis with consideration for repeat biopsy as the condition evolves. The initial clinical impression may not always correlate with the pathologic diagnosis, and conversely, only 61% of cases permit blinded diagnosis behind the microscope, emphasizing the importance of clinicopathologic correlation and collaboration with the dermatopathologist [4].

Psoriasis, which is a chronic autoimmune disorder, is characterized by erythematous scaling plaques with a predilection for regions such as the elbows and knees [2]. Erythrodermic psoriasis is a relatively rare form of psoriasis but represents a fairly common etiology for erythrodermic phenotypes. Other clinical variants of psoriasis include guttate, plaque-type, and pustular.

Erythrodermic psoriasis is characterized by erythema that is often exfoliative involving greater than 75% of the body surface area. Erythrodermic psoriasis may be accompanied by fever and malaise and is associated with significant morbidity, including increased rates of high-output congestive heart failure and sepsis. The onset is highly variable; it may occur acutely or persist chronically with recurrent relapses. It can serve as an initial presentation or emerge in longstanding disease [8-9].

Classic pathology findings on skin biopsy can help to establish the diagnosis of psoriasis. Typical pathologic changes include epidermal acanthosis, confluent parakeratosis, and uniform club-shaped elongation of the rete ridges with a thin suprapapillary plate. Collections of neutrophils may also be seen in either the lower layers of the epidermis (spongiform pustules of Kogoj), the stratum corneum (Munro’s microabscesses), or both [2, 9].

Various treatments for psoriasis exist, including topical therapies such as glucocorticoids and vitamin D derivatives, ultraviolet light therapy, and systemic agents that target key players in well-defined immune pathways [2]. Given the increased morbidity in erythrodermic psoriasis, prompt diagnosis and treatment are imperative.

During our patient’s initial workup to exclude the Sezary syndrome variant of cutaneous T-cell lymphoma (another cause of erythroderma), our patient was discovered to have monoclonal B-cell lymphocytosis. Monoclonal B-cell lymphocytosis is characterized by a small, clonal B-cell populations (usually CD5+, CD19+, CD20+, CD23+) detected in the peripheral blood [10]. Originally identified in 2005 as a lab abnormality seen in otherwise healthy individuals, monoclonal B-cell lymphocytosis has gained increasing attention due to its potential to progress to chronic lymphocytic leukemia [3].

Monoclonal B-cell lymphocytosis can be classified as low-count or high-count subtypes based on the cut-off of 0.5 x 10^9^ clonal B cells/liter [10]. This cut-off point is clinically important as high-count monoclonal B-cell lymphocytosis has a much higher association with progression to chronic lymphocytic leukemia. High-count monoclonal B-cell lymphocytosis is considered a premalignant syndrome, while low-count monoclonal B-cell lymphocytosis may be related to aging or chronic antigenic stimulation [3]. Furthermore, patients with low-count monoclonal B-cell lymphocytosis do not have an increased rate of progression to chronic lymphocytic leukemia.

Management of patients with monoclonal B-cell lymphocytosis is based on the patient’s B-cell lymphocyte count. Due to the exceedingly low risk of progression to chronic lymphocytic leukemia in low-count monoclonal B-cell lymphocytosis, patients require no specialized follow-up. Patients with high-count monoclonal B-cell lymphocytosis, however, are recommended for annual follow-up with a hematologist to assess clinical progression to chronic lymphocytic leukemia.

While psoriasis may occur in patients with cancer, it is not considered a paraneoplastic disease associated with chronic lymphocytic leukemia or monoclonal B-cell lymphocytosis. To the best of our knowledge, an association between monoclonal B-cell lymphocytosis and psoriasis has not been previously reported. Our patient was diagnosed with monoclonal B-cell lymphocytosis at the time of presentation for erythrodermic psoriasis. Whether this association between psoriasis and monoclonal B-cell lymphocytosis is bona fide or coincidental remains to be determined. Monoclonal B-cell lymphocytosis has been increasingly diagnosed in the past ten years due to advances in flow cytometry and is 100 times more prevalent than chronic lymphocytic leukemia [10]. As the sensitivity of flow cytometry and the overall age of the general population advance, monoclonal B-cell lymphocytosis is likely to be increasingly seen and characterized; therefore, skin or autoimmune associations, as demonstrated in our patient, may be increasingly identified.

## Conclusions

The differential diagnosis of erythroderma includes various conditions, and the most important investigation is delving into a past history of cutaneous skin disease. Often times, a past history of psoriasis, eczema, or a drug eruption is the most important clue to the onset of an erythrodermic phenotype. A skin biopsy is essential and may help to better classify the process. Psoriasis can present with erythroderma, and in some patients, the clinical features can mimic those of pityriasis rubra pilaris and vice versa. Monoclonal hematologic disorders have been increasingly diagnosed in the last decade and may be a precursor to chronic lymphocytic leukemia. Individuals with the high-count variant are at an increased risk of progression to a chronic lymphocytic leukemia compared to those with the low-count subtype. Our patient’s diagnosis of monoclonal B-cell lymphocytosis occurred concurrently with a severe, new, and atypical development of erythrodermic psoriasis. Whether the two conditions occurred independently as a coincidental event or share a related pathogenesis remains to be established, and additional reports may be helpful to elucidate if a connection between these conditions exists.
